# Functionalized Bacterial Cellulose Bottlebrush‐Based Asymmetric Dressing for Effective Management of Wounds with Infection and Exudate

**DOI:** 10.1002/smsc.202300138

**Published:** 2023-11-20

**Authors:** Zifeng Yang, Feng Wang, Chenguang Shi, Junlong Huang, Ruijun Xu, Luna Quan, Yang Li, Qi Sun, Hui Wang, Rongkang Huang, Bingna Zheng, Yong Li

**Affiliations:** ^1^ Department of Gastrointestinal Surgery Department of General Surgery Guangdong Provincial People's Hospital (Guangdong Academy of Medical Sciences) Southern Medical University Guangzhou 510080 China; ^2^ Guangdong Cardiovascular Institute Guangdong Provincial People's Hospital Guangdong Academy of Medical Sciences Guangzhou 510080 China; ^3^ PCFM Lab School of Chemistry Sun Yat-sen University Guangzhou 510006 China; ^4^ School of Medicine South China University of Technology Guangzhou 510006 China; ^5^ Department of General Surgery (Colorectal Surgery) Guangdong Institute of Gastroenterology Biomedical Innovation Center Guangdong Provincial Key Laboratory of Colorectal and Pelvic Floor Diseases The Sixth Affiliated Hospital Sun Yat-sen University Guangzhou 510655 China; ^6^ Center of Accurate Diagnosis Treatment and Transformation of Bone and Joint Diseases The Eighth Affiliated Hospital Sun Yat-sen University Shenzhen 518000 China

**Keywords:** antibacteria, antireflux, asymmetric dressing, bacterial cellulose bottlebrushes, exudate absorption

## Abstract

Simultaneous infection and exudate management remain a great challenge in wound treatment. As one of the most promising approaches, current asymmetric dressings are basically prepared through electrospinning with different kinds of materials, and the interfacial interaction remains unexplored. Herein, the first single‐substrate‐based asymmetric dressing with a well‐entangled interface, large absorptive capacity, effective fluid reflux prevention, excellent antibacterial, and antipenetration properties is developed. The asymmetric dressing is composed of layered hydrophilic and hydrophobic bottlebrushes (sodium polyacrylate and polystyrene‐functionalized bacterial cellulose), which are assembled using stepwise vacuum filtration, accompanied by loading triclosan into the solution of hydrophilic bottlebrushes. The biocompatibility and feasibility of the asymmetric dressing pave the way in real wound management.

## Graphical abstract

A triclosan‐loaded asymmetric dressing with a hydrophilic top layer and a hydrophobic bottom layer, assembled using two types of functionalized nanofibers, polystyrene and sodium polyacrylate‐grafted bacterial cellulose, is capable of absorbing exudate, maintaining a dry biofluid interface and providing broad‐spectrum antibacterial properties.

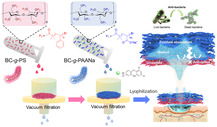

## 1 Introduction

**Figure 1:**
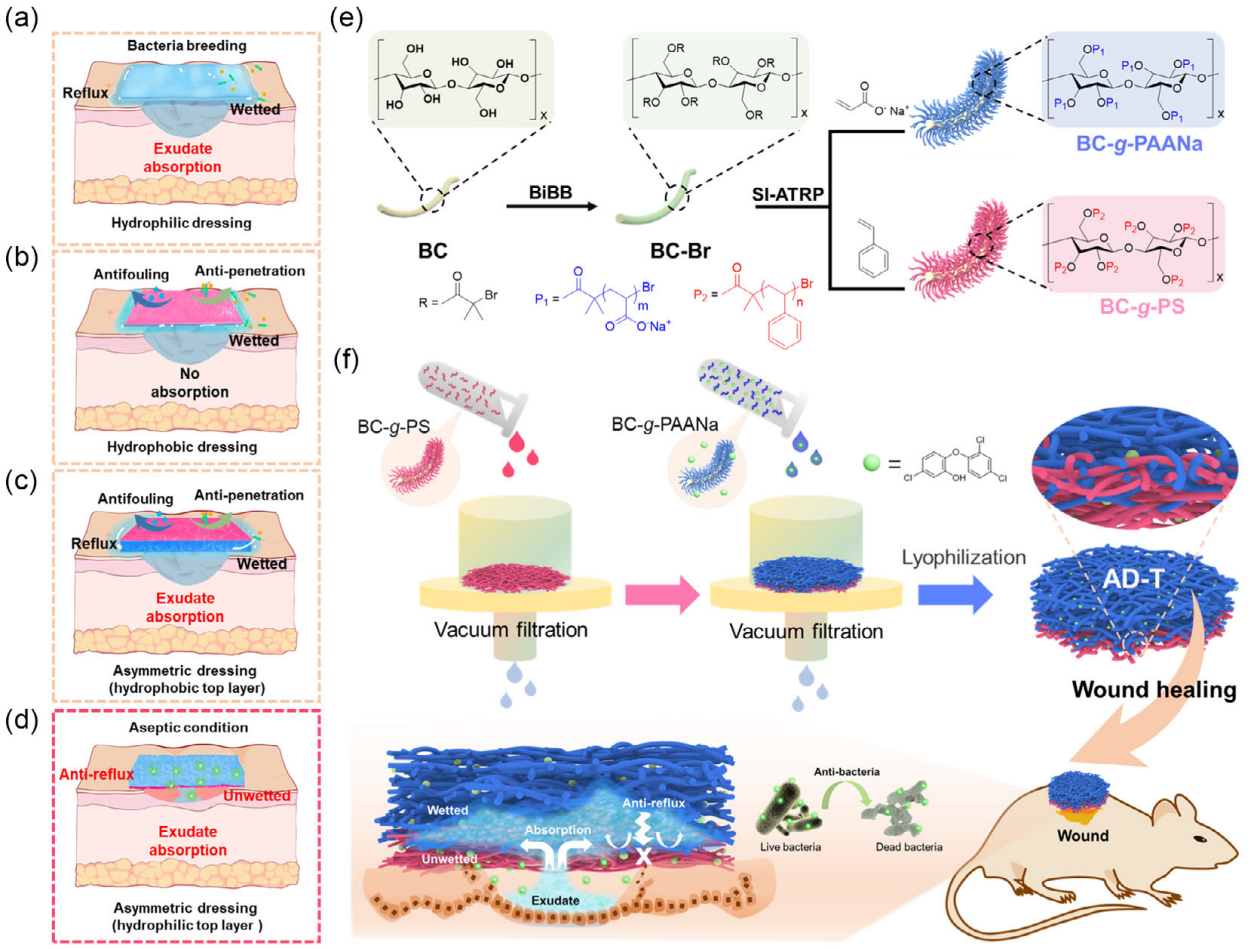
a) Hydrophilic dressings have a high absorptive capacity of exudate, but bacteria breeding and reflux are the main problems. b) Hydrophobic dressings can prevent fouling and bacteria penetration, but it lacks the ability to absorb exudate. c) Asymmetric dressing with a hydrophobic top layer possesses the qualities of exudate absorption, antifouling, and resistance to bacteria penetration, but still encounters reflux. d) Asymmetric dressings with a hydrophilic top layer have the properties of exudate absorption, antibacteria, and antireflux, which can keep the interface relatively dry and promote wound healing. e) Preparation of BC‐*g*‐PAANa and BC‐*g*‐PS by SI‐ATRP. f) Schematic illustration of the fabrication, structure, and properties of our AD‐T.

Infection and excessive exudate are major challenges in wound management. The presence of infection tends to exacerbate the exudate, while excessive exudate creates an environment conducive to bacterial growth, creating a vicious cycle.^[^ ^1^, ^2^, ^3^ ^]^ To effectively manage such wounds, a relatively dry state and aseptic condition are necessary.^[^ ^4^, ^5^ ^]^ Hydrophilic single‐layer dressings, such as cotton gauze, foams, and hydrogels, can rapidly absorb exudate and physically isolate the wound.^[^ ^6^, ^7^, ^8^ ^]^ However, limitations arise due to biofluid wetting of the interface and excessive reflux of exudate (**Figure** **1**a).^[^ ^4^ ^]^ On the other hand, hydrophobic single‐layer dressings demonstrate excellent antifouling and antibacterial barrier properties for physical isolation.^[^ ^9^, ^10^, ^11^ ^]^ Nevertheless, their inability to absorb exudate can result in wound impregnation (Figure 1b, Table S1, Supporting Information). Asymmetric dressings with a hydrophobic top layer and a hydrophilic bottom layer combine the advantages. However, as the wound is covered by the wet hydrophilic layer, this design causes further problems of interfacial biofluid wetting and wound impregnation (Figure 1c, Table S1, Supporting Information).^[^ ^12^, ^13^, ^14^ ^]^ Therefore, developing dressings that can rapidly remove the excessive exudate, maintain the dry state of the biofluid interface, and provide highly effective antibacterial properties is of great scientific importance and clinical value.

To simultaneously meet the above requirements, the design and construction of another type of multifunctional asymmetric dressing with a hydrophilic top layer and a hydrophobic bottom layer (AD), featuring a hydrophobic layer in contact with the wound, represents a promising solution (Figure 1d).^[^ ^4^, ^15^ ^]^ The hydrophobic layer has the ability to keep the biofluid interface dry and prevent the reflux of exudate. Meanwhile, the hydrophilic layer has strong absorption for fluid, allowing the absorption and subsequent drainage of fluid through the thin hydrophobic layer. In practice, current bottom‐hydrophobic asymmetric wound dressings are composed of various materials (e.g., inorganic and organic,^[^ ^16^ ^]^ natural and artificial,^[^ ^17^, ^18^, ^19^ ^]^ fiber and particle^[^ ^20^ ^]^) and prepared through electrospinning and laser beam (Table S3, Supporting Information). The diverse building blocks may have problems in interfacial compatibility and processability. These problems could be circumvented by single‐substrate‐based dressings. However, adopting the appropriate technology to achieve the opposite functional modification of a single substrate remains a challenge. To the best of our knowledge, there has not been a single substrate‐based asymmetric dressing reported.

Here, a triclosan‐loaded asymmetric dressing (AD‐T) is developed by integration of antibacterial agent with functionalized bacterial cellulose bottlebrushes exhibiting diverse hydrophilicity (Figure 1e,f). The abundant hydroxyl groups on the surface of BC are conducive to chemical modification, and hydrophilic and hydrophobic polymer hairs can be controllably grafted from BC by surface‐initiated atom transfer radical polymerization (SI‐ATRP).^[^ ^7^, ^21^, ^22^, ^23^ ^]^ The as‐obtained polystyrene grafted BC (BC‐*g*‐PS) and sodium polyacrylate grafted BC (BC‐*g*‐PAANa) are used as hydrophobic and hydrophilic structural units, respectively (Figure 1e). Benefiting from the same 1D flexible BC skeleton and well‐dispersed state in suitable dispersing reagents, BC‐*g*‐PS and BC‐*g*‐PAANa nanofibers can form entanglements during the process of stepwise vacuum filtration, thus producing AD with firmly bounded interlayer and ideal mechanical properties. Meanwhile, the interconnected pores derived from the stacking of nanofibers provide unobstructed channels for biofluid flow. To further enhance the antibacterial performance of AD, triclosan, a hydrophobic small‐molecule antibacterial agent is incorporated during the vacuum filtration, resulting in AD‐T with effective antibacterial performance. The asymmetric wettability of the two layers enables our AD‐T to integrate incompatible functions: the hydrophilic BC‐*g*‐PAANa layer shows excellent absorptive performance toward the fluid, while the hydrophobic force of the BC‐*g*‐PS layer can prevent the reflux of the fluid (Figure 1f). Furthermore, the biocompatibility and prohealing ability of AD‐T are substantiated by both in vitro and in vivo tests, thus providing a new avenue for the design of multifunctional dressings.

## 2 Results and Discussion

### 2.1 Preparation and Physicochemical Characterizations

**Figure 2:**
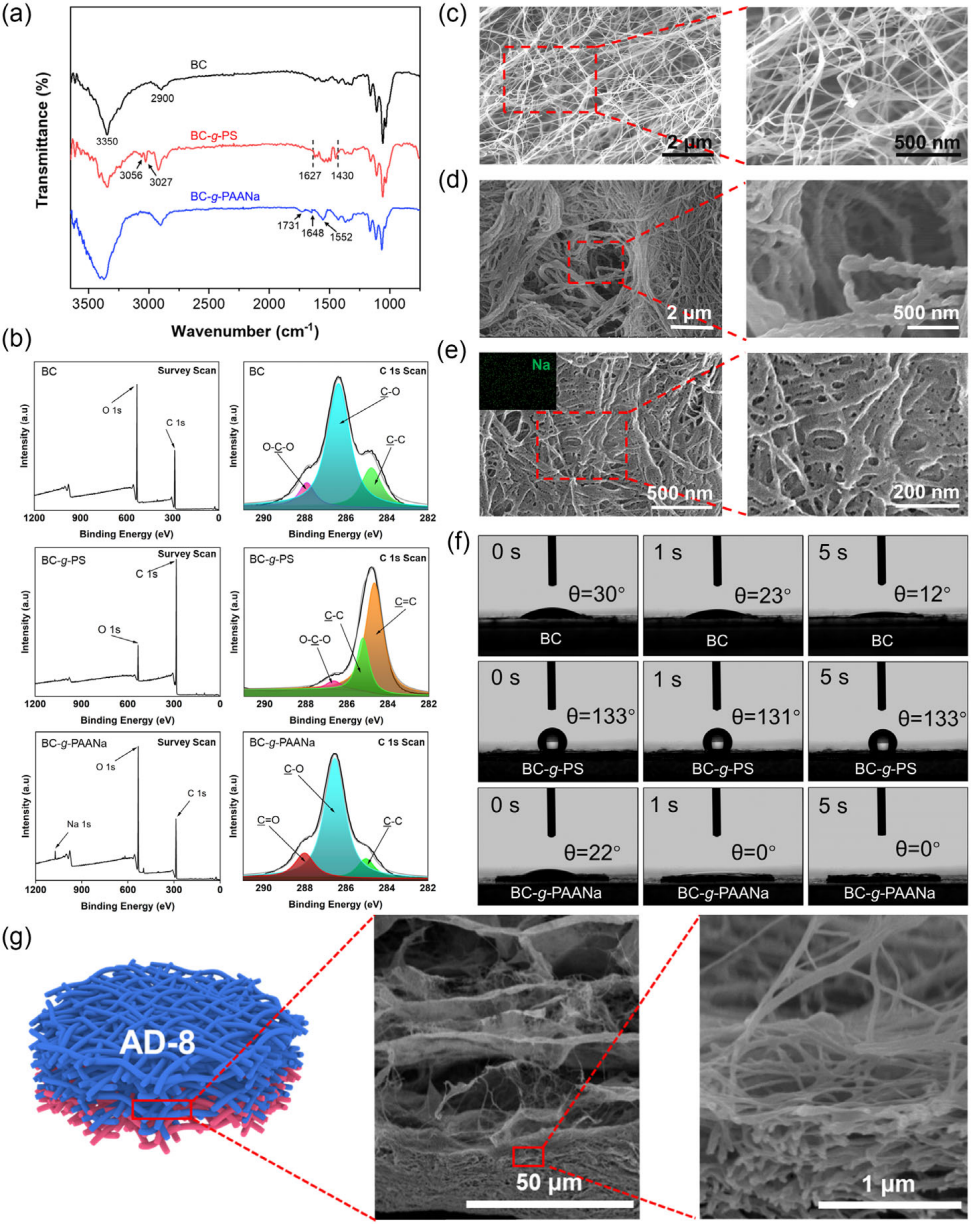
a) FTIR spectra of BC, BC‐*g*‐PS, and BC‐*g*‐PAANa. b) XPS spectra and high‐resolution C 1_*S*_ XPS spectra of BC, BC‐*g*‐PS, and BC‐*g*‐PAANa. c–e) Scanning electron microscopy (SEM) images of BC (c), BC‐*g*‐PS (d), and BC‐*g*‐PAANa (e). The inset of (e) shows the element mapping of Na. f) Water contact angles of the BC, BC‐*g*‐PS, and BC‐*g*‐PAANa films at different times. g) SEM images of AD‐8 at the interface between BC‐*g*‐PS and BC‐*g*‐PAANa.

Hydrophobic polystyrene (PS) and hydrophilic sodium polyacrylate (PAANa) are successfully grafted from BC to prepare BC‐*g*‐PS and BC‐*g*‐PAANa after the SI‐ATRP, respectively. In the Fourier transform infrared (FTIR) spectra (**Figure** **2**a), the peaks at 2900 cm^−1^ are attributed to the C–H stretching vibration of BC, and the broad peaks at ≈3350 cm^−1^ are mainly attributed to the vibration of hydroxyl groups from BC.^[^ ^24^, ^25^ ^]^ For BC‐*g*‐PS, the peaks at 3056 and 3027 cm^−1^ demonstrate the C–H stretching vibration of the phenyl groups of the grafted PS^[^ ^26^ ^]^, and a series of peaks at 1430–1627 cm^−1^ are attributed to the C–C stretching vibration of the benzene skeleton.^[^ ^27^, ^28^, ^29^ ^]^ For BC‐*g*‐PAANa, the peaks at 1731, 1648, and 1552 cm^−1^ represent the C=O stretching vibration.^[^ ^30^, ^31^, ^32^ ^]^

Compared with the X‐ray photoelectron spectroscopy (XPS) spectra of BC (Figure 2b), BC‐*g*‐PS shows a prominent peak of C 1*s* with a binding energy of 284.65 eV, which is attributed to C=C bonds, indicating the successful grafting of PS.^[^ ^33^, ^34^ ^]^ In the survey scan of BC‐*g*‐PAANa, a peak of Na 1*s* appeared. The peak at 288.03 eV is attributed to C=O bonds of the grafted PAANa hairs.^[^ ^35^, ^36^ ^]^ As shown in Figure 2d,e, the nanofibers of BC‐*g*‐PS and BC‐*g*‐PAANa have a rougher surface and a larger diameter as compared to pristine BC (Figure 2c).^[^ ^30^, ^37^ ^]^ Part of the grafted polymer chains fill the nanopores formed by the stacking of nanofibers. The inset of Figure 2e shows the element mapping of Na for BC‐*g*‐PAANa. The uniformly distributed green dots representing Na demonstrate the uniform distribution of grafted PAANa.

After functionalization, the contact angles of BC, BC‐*g*‐PS, and BC‐*g*‐PAANa prove the significant differences in water affinity (Figure 2f).^[^ ^27^ ^]^ The contact angle of BC‐*g*‐PAANa film is 0° at 1 s, showing its superhydrophilicity. In contrast, the BC‐*g*‐PS film maintains a contact angle of ≈130°, confirming the successful hydrophobic functionalization (Figure 2f). BC, BC‐*g*‐PS, and BC‐*g*‐PAANa exhibit different dispersion phenomena in deionized water (Figure S1a, Supporting Information). The hydrophilic BC and BC‐*g*‐PAANa can be uniformly dispersed in water, and the fluids present milky white and semiturbid state. In contrast, BC‐*g*‐PS, which is hydrophobic, rapidly aggregates and completely floats on the top layer within 15 s after shaking (Figure S1b, Supporting Information). Furthermore, the BC‐*g*‐PS and BC‐*g*‐PAANa films are simultaneously placed into deionized water. The BC‐*g*‐PAANa film sinks to the bottom within 10 s due to its rapid and extensive water absorption. In contrast, the BC‐*g*‐PS film floats on the deionized water (Figure S2a, Supporting Information). The swelling ratio of the hydrophilic BC film (0.65 wt%, lyophilization after 4 h vacuum filtration) is ≈2000%, similar to that reported in the literature.^[^ ^38^ ^]^ The BC‐*g*‐PAANa film (8 mg mL^−1^, lyophilization after 4 h vacuum filtration) has a higher swelling ratio (≈5300%) and a faster water absorption than the BC film. In contrast, the swelling ratio of the BC‐*g*‐PS film (0.2 mg mL^−1^, lyophilization after 5 min of vacuum filtration) is essentially 0 (Figure S2b, Supporting Information).

The preparation of AD consists of two main steps, vacuum filtration and lyophilization (Figure S3, Supporting Information). To ensure the integrity and flatness of the samples, the selection of suitable solvents is crucial (Figure S4, Supporting Information). DMF enables the uniform dispersion of BC‐*g*‐PS and BC‐*g*‐PAANa, leading to a flat sample after vacuum filtration. However, the as‐obtained AD sample is significantly shrunken after lyophilization (Figure S4a, Supporting Information). The use of THF and DMF as solvents for BC‐*g*‐PAANa and BC‐*g*‐PS during vacuum filtration leads to a flat sample due to good dispersion, but the final sample becomes crumbled and fragile (Figure S4b, Supporting Information). The integrity and flatness after vacuum filtration can also be realized by choosing deionized water and DMF as solvents for BC‐*g*‐PAANa and BC‐*g*‐PS, respectively. Unfortunately, the final AD sample experiences detachment of the powder of BC‐*g*‐PS layer and subsequent separation of the two layers after lyophilization (Figure S4c, Supporting Information). Using deionized water as a solvent, vigorously shaken BC‐*g*‐PS is quickly filtrated and the obtained sample is smooth. However, the freeze‐dried AD sample exhibits complete separation (Figure S4d, Supporting Information). When BC‐*g*‐PS is dispersed in DMF or THF, and BC‐*g*‐PAANa is dispersed in the 50% v/v aqueous DMF or THF solution, the integrity and flatness of the final sample can be ideally maintained without shrinkage, fragility, delamination, or separation (Figure S4e,f, Supporting Information). This is ascribed to the dispersive effects of our 1D polymer bottlebrushes in different solvents. With the formation of the hydrophobic BC‐*g*‐PS layer by vacuum filtration, BC‐*g*‐PAANa solution is subsequently added. As both BC‐*g*‐PS and BC‐*g*‐PAANa are well dispersed in 50% v/v aqueous THF solution (Figure S5, Supporting Information), the two kinds of polymer bottlebrushes can interdiffuse at the interface, and these flexible and stretched nanofibers can entangle with each other, eventually resulting in the AD with a firmly bounded interlayer. A typical asymmetric morphology with a relatively loose BC‐*g*‐PAANa hydrophilic layer and a dense BC‐*g*‐PS hydrophobic layer can be observed. Meanwhile, the entangled structure of hydrophilic and hydrophobic bacterial cellulose is observed at the interface (Figure 2g). With such uniform dispersion and high integrity, the tensile strength at break and Young's modulus of AD‐7 (3.2, 13.94 MPa) is comparable to those of BC (3.4, 16.92 MPa) (Figure S6, Supporting Information).

### 2.2 Fluid Absorption and Antireflux Performances

**Figure 3:**
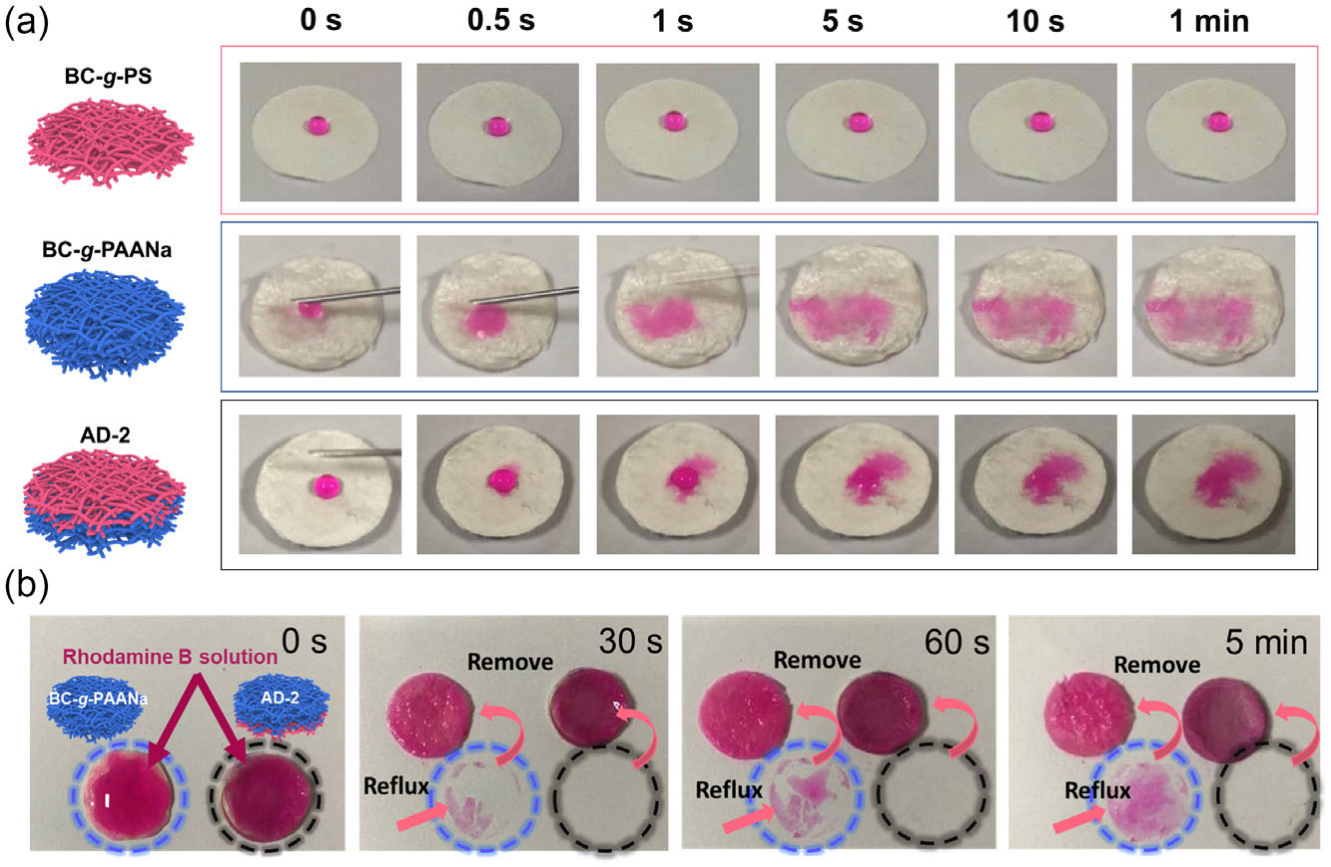
a) Digital photos of the absorption performance of BC‐*g*‐PS, BC‐*g*‐PAANa, AD‐2. b) Antireflux performance of BC‐*g*‐PAANa and AD‐2 at different times.

Rhodamine B aqueous solution was dropped on BC‐*g*‐PS film, BC‐*g*‐PAANa film, and AD‐2 (with the BC‐*g*‐PS layer facing up), respectively, to evaluate their fluid absorption performance (**Figure** **3**a). The droplet on the BC‐*g*‐PS film is maintained throughout without spreading, confirming the stable hydrophobicity of BC‐*g*‐PS. Due to its hydrophilicity, the droplet on the BC‐*g*‐PAANa film diffuses rapidly within about 0.5 s, and the swelling ratio reaches to ≈4300% (Figure S7, Supporting Information), verifying its excellent absorption capability. With such a high absorption tendency, when the solution is dropped on AD‐2, it can be pumped from the channels of the thin hydrophobic layer to the hydrophilic layer.^[^ ^39^ ^]^ To further verify the influence of BC‐*g*‐PS layers with different thicknesses on the water absorption performance and change in contact angles of ADs, samples with the same size of hydrophilic layer (i.e., 2 mm) were prepared. The thinner the hydrophobic BC‐*g*‐PS layer is, the faster the water can be absorbed.^[^ ^40^, ^41^ ^]^ When the hydrophobic layer is increased to 60 μm (AD‐5), the size and contact angles (≈130°) of the water droplets maintain unchanged, indicating no absorption by the hydrophilic layer (Figure S8–S9, Supporting Information). This may be related to the strong hydrophobic force provided by the densely packed hydrophobic layer.^[^ ^40^, ^42^ ^]^ Furthermore, AD‐6 with a much thicker hydrophobic layer is also tested (Figure S10, Supporting Information). Ascribed to the large thickness and dense stacking of nanofibers for the hydrophobic layer, the water droplet is still not be absorbed into the hydrophilic layer, even when the 5 mm‐thick BC‐*g*‐PAANa layer with higher water absorption is used. By artificially making full thickness holes in the BC‐*g*‐PS layer (0.5 mm in diameter), the hydrophilic layer can immediately absorb the droplets through these holes. Therefore, the thickness of the hydrophobic layer and the pore size are crucial in controlling the fluid pumping behavior.^[^ ^43^, ^44^ ^]^

Due to the barrier property of the hydrophobic BC‐*g*‐PS layer, the anti‐reflux performance of AD‐2 was tested using a simple model (Figure S11, Supporting Information). The BC‐*g*‐PAANa film (2 mm) and AD‐2 saturated with aqueous Rhodamine B solution are placed on filter paper. After different periods of time, the samples are gently removed. A clear red staining of the paper can be observed under the BC‐*g*‐PAANa film, and the area of staining increases with time (60 s and 5 min). In contrast, the underside of AD‐2 is clean without staining even after 5 min (Figure 3b). This means that the hydrophobic layer is capable of preventing liquid reflux.^[^ ^4^, ^19^ ^]^

### 2.3 Antibacterial Performances

Triclosan is a highly potent and broad‐spectrum antibacterial agent with low effective concentration and rare drug resistance. Its antibacterial principle is mainly the bacterial target allosteric, which causes bacterial fluid outflow and inactivation or degradation of related enzymes.^[^ ^45^, ^46^ ^]^ Therefore, triclosan‐loaded medical materials, such as sutures, have been used clinically.^[^ ^47^ ^]^ To facilitate the antibacterial property of our AD, triclosan was directly introduced during filtration of hydrophilic BC‐*g*‐PAANa layer, resulting in AD‐T (Figure 1f). Due to the interconnected nanopores and hydrophobic interaction with BC‐*g*‐PS, triclosan can be loaded into the AD.

**Figure 4:**
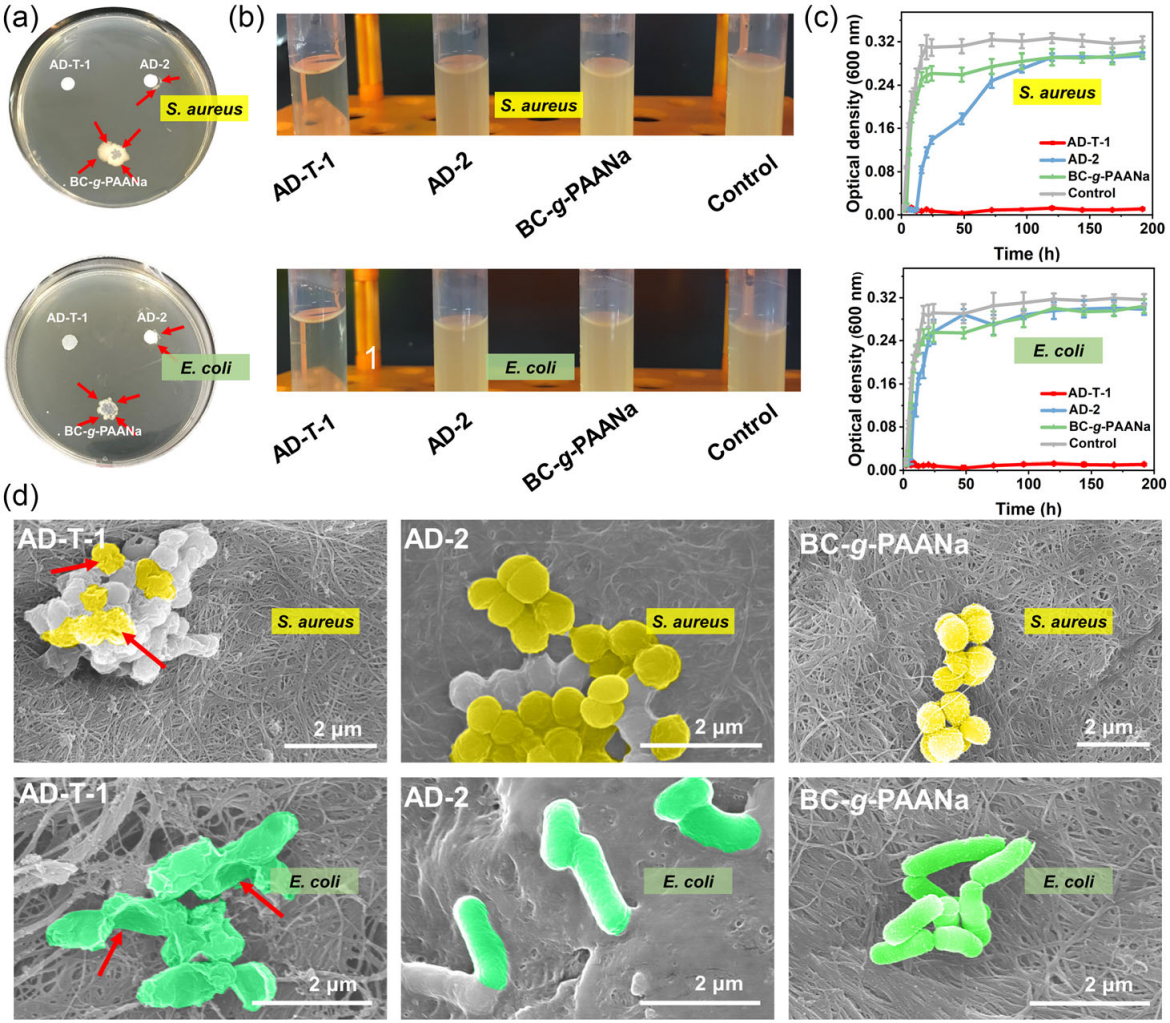
a,b) Digital photos of the bacterial penetration (a) and contact activity (b) and c) growth curves in the presence of culture solution, BC film, AD‐2, and AD‐T‐1 (*n* = 3). d) SEM images of bacterial adhesion of the AD‐T‐1, AD‐2, and BC‐*g*‐PAANa film.

*Staphylococcus aureus* and *Escherichia coli* were used for a series of antibacterial assays. *S. aureus* and *E. coli* bacterial suspensions were dropped onto AD‐T‐1, AD‐2, and BC‐*g*‐PAANa film to evaluate the bacterial penetration performance (**Figure** **4**a). The perimeter of the BC‐*g*‐PAANa film alone is covered with colonies, and even the growing bacteria climbs to cover the surface of the samples, consistent with the fact that BC‐*g*‐PAANa itself has no antibacterial component and swells after absorbing the bacterial solution, thus widening the internal fiber gap to facilitate bacterial penetration, crawling, and colonization (Figure S12a, Supporting Information). As for the AD‐2 sample, there are scattered colonies around the area covered by AD, significantly less than that around the BC‐*g*‐PAANa films, mainly due to the antipenetration of the hydrophobic BC‐*g*‐PS layer at the bottom (Figure S12b, Supporting Information).^[^ ^11^, ^48^ ^]^ In contrast, no colonies are seen around the area covered by AD‐T‐1, indicating the strong antibacterial activity of AD‐T‐1 during penetration (Figure S12c, Supporting Information).

In the bacterial contact assay, the samples of experimental group, consisting of AD‐T‐1, AD‐2, and BC‐*g*‐PAANa film, were immersed in a culture solution containing *S. aureus* and *E. coli*, and the control group consisted of the same concentration of bacteria without sample (Figure 4b). The optical density (OD_600_) of the bacterial culture was measured at different times to plot the growth curve. As shown in Figure 4c, the OD_600_ value of AD‐2, BC‐*g*‐PAANa film, and the control group starts to increase significantly after the first 6 h and reaches the peak within 24 h. In contrast, the OD_600_ value of the AD‐T‐1 group shows no increase after 192 h of culture, indicating that the bacteria were completely killed. At the same time, the AD‐T‐1, AD‐2, and BC‐*g*‐PAANa film were removed after 24 h of culture and then fixed and lyophilized (Figure 4d). The killed bacteria in the AD‐T‐1 group demonstrates an obvious morphology deformation, collapse, and disintegration. In contrast, the bacteria on the surface of the AD‐2 and BC‐*g*‐PAANa film still maintains a regular and complete shape.^[^ ^49^ ^]^

### 2.4 Cytocompatibility

**Figure 5:**
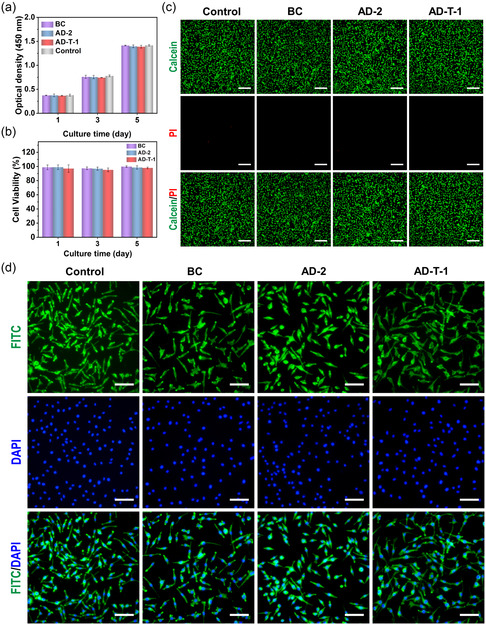
a) OD_450_ value and b) cell viability of L929 cells on different samples after 1, 3, and 5 days of culture (*n* = 3). c) Live/dead cell staining images of BC film, AD‐2, and AD‐T‐1 (scale bar: 200 μm). d) Cytoskeleton staining images of BC film, AD‐2, and AD‐T‐1 (scale bar: 100 μm).

Good biocompatibility is essential for a dressing, and it should be ensured that the dressing does not induce cytotoxicity.^[^ ^4^, ^16^, ^50^ ^]^ The BC film, AD‐2, and AD‐T‐1 were soaked in mouse fibroblast (L929 cell) solution and cultured for 5 days. Cell viability was determined using the Cell Counting Kit‐8 reagent (CCK‐8). The OD_450_ values show that the proliferation activity of L929 cells in each group gradually increased with the increasing culture time (**Figure** **5**a). The OD_450_ values of the BC, AD‐2, and AD‐T‐1 groups are basically the same as those of the control group on day 1, 3, and 5. Furthermore, the cell viability of the BC film, AD‐2, and AD‐T‐1 groups essentially reaches 100% on day 1, 3, and 5, indicating their good cytocompatibility (Figure 5b). Furthermore, the results of live/dead cell staining experiments show that most of the L929 cells in the BC film, AD‐2, and AD‐T‐1 groups are green (green and red cells are live and dead cells, respectively), which is similar to the control group (Figure 5c). For a more visual indication of cell viability, immunofluorescence staining of the L929 cells grown on the surface of BC film, AD‐2, and AD‐T‐1 was performed using DAPI and Actin Tracker Green. Figure 5d shows normal cytoskeletal (green) and nuclear (blue) morphology in all experimental samples and controls. The cells are spindle shaped with a uniform density of cell layers. To further evaluate the hemocompatibility, the hemolysis activity of BC, AD‐2, and AD‐T‐1 dispersions with various concentrations (625, 1250, and 2500 μg mL^−1^) was tested. After centrifugation, photographs of the supernatant of all experimental groups, the negative PBS group, and the positive Triton X‐100 group were taken. The AD‐T‐1 group presents a light yellow color similar to the negative PBS group, while the positive Triton X‐100 group appears bright red (Figure S13, Supporting Information). As shown in Figure S14, Supporting Information, the hemolysis ratios of BC, AD‐2, and AD‐T‐1 are less than 1% at all concentrations, demonstrating the good hemocompatibility.^[^ ^51^, ^52^ ^]^

### 2.5 In vivo Prohealing Evaluation

**Figure 6:**
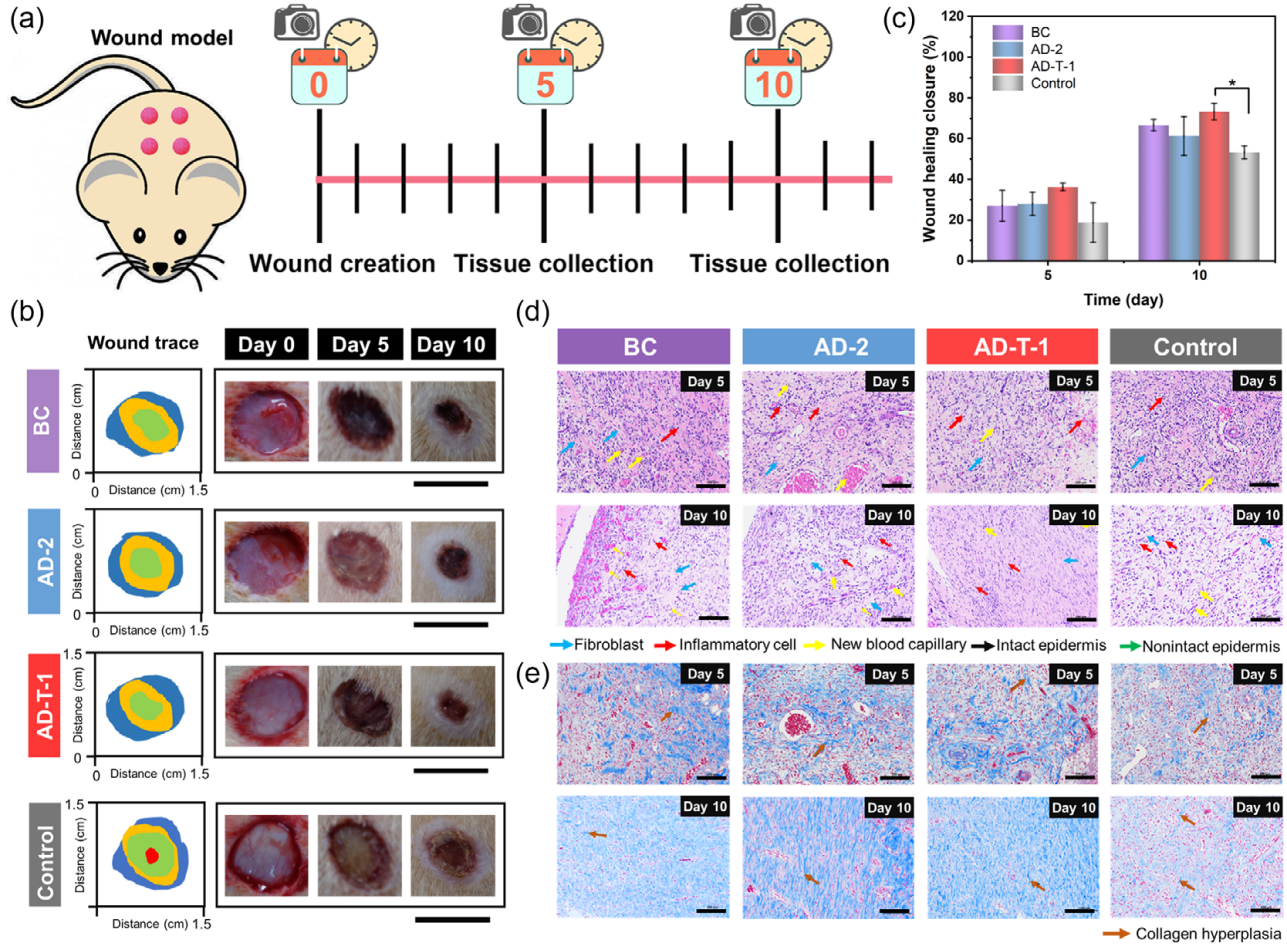
a) In vivo healing effect of BC, AD‐2, and AD‐T‐1 on the acute full‐thickness wound model and tissue collection on day 5 and 10. b) Digital photos of wound healing and wound trace after treatment with BC, AD‐2, and AD‐T‐1; scale bars: 10 mm. c) Statistics analysis of the wound closure rates on day 5 and 10 (*n* = 3, * *p* < 0.05). d) HE staining and e) Masson staining on day 5 and 10 of the newly regenerated skin tissue after treatment with BC, AD, and AD‐T‐1; scale bars: 100 μm.

A rat back wound model covered with BC film, AD‐2, and AD‐T‐1 was used to observe the wound status (e.g., dermal redness, swelling, and allergy) and wound healing rate (**Figure** **6**a). All the samples in the experimental groups healed faster than the control group within 10 days, which may be related to the physical barrier effect provided by the dressings to prevent infection by foreign bacteria (Figure 6b). Figure 6c provides a visualized trend of wound healing. In addition, on day 5, the presence of a yellow sheet‐like substance on the surface of the wounds in the control group could be an indication of infection, which would affect the healing rate. Due to its effective and sustained bactericidal ability, the wounds covered with AD‐T‐1 have the fastest healing rate. In addition, no redness or swelling was observed in the wounds covered with BC film, AD‐2, and AD‐T‐1 during the healing period, demonstrating their good biocompatibility.

To better assess the effect of BC film, AD‐2, and AD‐T‐1 on the inflammatory response during tissue healing, the healing epidermis was subjected to histological and immunohistochemical analysis on day 5 and 10, focusing on inflammatory cell infiltration, new capillary regeneration, fibroblast proliferation, and collagen deposition.^[^ ^7^, ^14^, ^53^ ^]^ The control group has a more severe inflammatory response with loose connective tissue and more hemorrhages than the other groups on day 5 (Figure 6d). At the same time, new capillary formation, fibroblast proliferation, and local congestion are observed in all groups. On day 10, the newly formed epidermis in the control group exhibits a thin structure with a significant presence of inflammatory exudates and infiltrated inflammatory cells, while the connective tissue appears to be loosely arranged. In contrast, in the AD‐T‐1 group, despite the incomplete nature of the epidermis, a significant number of tightly organized connective tissues and fibroblasts are observed. Furthermore, the amount of inflammatory exudate and inflammatory cells is comparatively lower than that in the other three groups. Compared with the AD‐T‐1 group, the AD‐2 and BC film groups show a greater presence of inflammatory exudate and looser connective tissue beneath the epidermis, together with a greater number of fibroblasts and new capillaries. The tissue was then subjected to Masson's staining to assess the formation and deposition of collagen. On day 5, collagen deposition is sparsely distributed in all groups, with the control group showing the lower level of collagen deposition. As the wound heals, collagen deposition becomes denser in the BC film, AD‐2, and AD‐T‐1 groups, accompanied by improved formation of collagen bundles compared to day 5 (Figure 6e). In contrast, the control group shows dysplasia, with broken collagen bundles and sparsely distributed areas of blue staining.

## 3 Conclusion

We have synthesized the first single‐substrate‐based asymmetric dressing by functionalizing BC with hydrophobic PS and hydrophilic PAANa via SI‐ATRP. The layer thickness and interface entanglement can be easily tuned via polymer bottlebrush amount and solvent selection. The bottom hydrophobic layer is demonstrated essential for rapid absorption and fluid reflux prevention. With optimized layer thickness, well‐entangled interface, and interconnected pores, the asymmetric dressing exhibits balanced absorption/antireflux properties and robust mechanical properties. After loading triclosan, AD‐T exhibits effective antibacterial properties, good biocompatibility, and prohealing ability in both in vitro and in vivo experiments. Overall, our single‐substrate‐based asymmetric dressing not only offers an effective wound management approach, but also provides a model platform for interface interaction investigation.

## Conflict of Interest

The authors declare no conflict of interest.

## Supporting information

Supplementary Material

## Data Availability

The data that support the findings of this study are available from the corresponding author upon reasonable request.
