# Supplementary material for: Functionalized Bacterial Cellulose Bottlebrush‐Based Asymmetric Dressing for Effective Management of Wounds with Infection and Exudate
Source: Small Sci. 2023 Nov 20;3(12):2300138. doi: 10.1002/smsc.202300138 (PMC11936004; doi:10.1002/smsc.202300138)
Supplement: Supplementary file 1 — Supplementary Material [file SMSC-3-2300138-s001.pdf]

Supporting Information

Functionalized Bacterial Cellulose Bottlebrush-Based Asymmetric Dressing for Effective Management of Wounds with Infection and Exudate

Zifeng Yang, Feng Wang, Chenguang Shi, Junlong Huang, Ruijun Xu, Luna Quan, Yang Li, Qi Sun, Hui Wang *, Rongkang Huang *, Bingna Zheng *, Yong Li *

Z. Yang, F. Wang, J. Huang, Y. Li, Prof. Y. Li

Department of Gastrointestinal Surgery, Department of General Surgery

Guangdong Provincial People's Hospital (Guangdong Academy of Medical Sciences)

Southern Medical University

Guangzhou, 510080, China

E-mail: liyong@gdph.org.cn

F. Wang, Prof. Y. Li

Guangdong Cardiovascular Institute

Guangdong Provincial People's Hospital

Guangdong Academy of Medical Sciences

Guangzhou, 510080, China

C. Shi, L. Quan

PCFM Lab

School of Chemistry

Sun Yat-sen University

Guangzhou 510006, China

R. Xu, Q. Sun

School of Medicine

South China University of Technology

Guangzhou, 510006, China

Prof. H. Wang, R. Huang

Department of General Surgery (Colorectal Surgery)

Guangdong Institute of Gastroenterology

Biomedical Innovation Center

Guangdong Provincial Key Laboratory of Colorectal and Pelvic Floor Diseases

The Sixth Affiliated Hospital

Sun Yat-sen University

Guangzhou 510655, China

E-mail: wang89@mail.sysu.edu.cn

E-mail: huangrk3@mail.sysu.edu.cn

B. Zheng

Center of Accurate Diagnosis

Treatment and Transformation of Bone and Joint Diseases

The Eighth Affiliated Hospital

Sun Yat-sen University

Shenzhen 518000, China

E-mail: zhengbn3@mail.sysu.edu.cn

Experimental Section

Materials and Chemicals

Bacterial cellulose water-dispersion (BC, 0.65 wt.%) was purchased from Guilin Qihong Technology Co., Ltd. (China). Triethylamine (TEA, 99%), 2-bromoisobutyryl bromide (BiBB, 98%), *N,N,N,N,N*-pentamethyldiethylenetriamine (PMDETA, 99%), copper (II) bromide ($CuBr_2$, > 99%), styrene, sodium acrylate, sodium chloride, ascorbic acid (AAc, ≥ 99%), tetrahydrofuran (THF), methanol (99.8%), ethanol (99.8%) and phosphate buffered saline (PBS) were purchased from Shanghai Aladdin Biochemical Technology Co., Ltd. (China). Fetal bovine serum (FBS), Dulbecco's modified Eagle's medium (DMEM), and penicillin-streptomycin solution were purchased from Gibco Technologies Co., Ltd. (USA). Cell counting kit-8 (CCK-8) was purchased from Sigma-Aldrich (Shanghai) Trading Co., Ltd. (China). The live/dead cell viability kit (Calcein-AM/PI) was purchased from Shanghai Xinyu Biotechnology Co., Ltd. (China). Calcein AM/PI Double Staining Kit was purchased from Dalian Meilun Biotech Co., Ltd. (China). Staphylococcus aureus (*S. aureus*, ATCC 6538) and Escherichia coli (*E. coli*, ATCC 25922) were obtained from Guangdong Microbial Culture Collection Center. Deionized (DI) water was used in the experiment. All other chemicals were purchased from Sigma-Aldrich (Shanghai) Trading Co., Ltd. (China). Male Sprague Dawley (SD) rats (12 weeks, 200-240 g) were purchased from the Laboratory Animal Center of Sun Yat-sen University (Guangzhou, China). All rats were fed and tested following laboratory rules and guidelines. Histological analysis was performed by Wuhan Servicebio Technology Co., Ltd. (China).

Preparation of BC-Br

The water in the BC dispersion was entirely replaced by DMF by centrifugation as a pretreatment. A total of 13.2 g pretreated BC, dispersed in 250 mL of DMF, was purged with nitrogen (N_2) for 30 min. The mixture was cooled to 0°C in an ice-water bath under an N_2 atmosphere, and 4 mL of TEA was added. 10 mL of DMF containing 4 mL of BiBB was added to the mixture dropwise in 30 min. The mixture was kept at 0°C under stirring for 2 h and then stirred at room temperature for 24 h. The initiator-functionalized BC-Br was obtained by washing thoroughly with ethanol and DI water

to remove residual reactants, and centrifuged at 12,000 rpm for 15 min. The final BC-Br was stored in a refrigerator at 4°C for later use.

Preparation of BC-*g*-PS

Typically, 4 g of BC-Br, 10 mL of styrene without inhibitor, 0.207 mL of PMDETA, and 50 mL of DMF were stepwise added in a Schlenk flask. The mixture was purged with N_2 for 30 min under stirring, and 0.065 g of $CuBr_2$ and 0.032 g of AA were added. The reaction was carried out at 75°C for 12 h. The reaction products were washed with DMF to remove residual reactants.[1] The as-obtained BC-*g*-PS was dispersed in THF for later use.

Preparation of BC-*g*-PAANa

According to the literature,[2, 3] 1 g of AANa, 2 g of NaCl, 4 g of BC-Br and 0.116 mL of PMDETA were dissolved in 60 mL of a 50% (v/v) aqueous methanol solution in a Schlenk flask. The mixture was purged with N_2 for 30 min, and 0.045 g of $CuBr_2$ and 0.015 g of AA were added. The reaction was carried out at 60°C for 24 h. The reaction products were washed with 0.1 M nitric acid, 0.1 M sodium hydroxide and DI water to remove the AANa monomer, free polymer and other residual reactants. The as-obtained BC-*g*-PAANa was dispersed in water/THF mixtures for further use.

Preparation of AD and AD-T

BC-*g*-PS was dispersed in THF and diluted to a concentration of approximately 0.2 mg mL^{-1}. BC-*g*-PAANa was fully dispersed in a 50% (v/v) aqueous THF solution, at a concentration of approximately 8 mg mL^{-1} with or without the addition of triclosan (1 mg mL^{-1}). With different filtration volumes and times, BC-*g*-PS and BC-*g*-PAANa layers of different thicknesses can be prepared, finally giving rise to ADs and AD-T with different thicknesses (Table S2).

Material characterization

Contact angle. The contact angle (CA) was measured using a Contact Angle System (KRUSS DSA100) at room temperature and ambient humidity. A 10 μL drop of deionized water was used as an indicator. Average CA values were obtained by measuring three samples.

X-ray photoelectron spectroscopy (XPS). X-ray photoelectron spectroscopy (XPS, Thermo-VG Scientific ESCALAB 250Xi) with a standard Al Ka X-ray source (1486.8 eV) was used to analyze the chemical structure of BC, BC-*g*-PS and BC-*g*-PAANa.

Field emission scanning electron microscopy (FE-SEM). The surface morphology, internal structure and elemental mapping of the samples were analyzed using a field emission scanning electron microscope (FE-SEM, Hitachi S-4800) manufactured in Japan. First, BC, BC-*g*-PS, BC-*g*-PAANa, and AD-8 were completely freeze-dried, and then all samples were adhered to a sample stage with conductive adhesive, and an optional cross-section scanning stage was used to observe the cross-sectional structures. Prior to observation, the samples were sprayed with gold twice, the second time with a 90° rotation of the stage. The sputtering current and time were 10 mA and 50 s, respectively.

Fourier transform infrared spectroscopy (FT-IR). The tested samples (BC, BC-*g*-PS, and BC-*g*-PAANa) were homogeneously mixed with potassium bromide (KBr) in a mass ratio of approximately 1:100, thoroughly ground, pressed into tablets using a manual tablet press and then baked and dried under an infrared lamp. FT-IR spectra were obtained using an FT-IR spectrometer (TENSOR 27, BRUKER, Germany).

Tensile test. Dumbbell-shaped specimens of BC and AD-7 (12 mm in length, 5 mm in width, 1 mm in thickness) were pressed for tensile testing. A universal mechanical testing machine (WD-5A, Guangzhou Experimental Instrument Factory, China) was used for the tests. A 100 N load cell was selected for the tests, and the strain rate was 15 mm min^{-1}.

Swelling ratio. The swelling ratios of BC, BC-*g*-PS, and BC-*g*-PAANa were calculated by the weight method. The samples were first filtrated into films and then completely lyophilized, and the dry weights (W_{dry}) were accurately measured. Each sample was placed in a centrifuge tube containing deionized water and tested at room temperature. At regular intervals, the samples were removed from the deionized water, and the wet weight (W_{wet}) was measured after gently removing excess water from the surface of the sample using filter paper. The swelling ratio is defined by the following formula:

$$\text{Swelling ratio (\%)} = (W_{wet} - W_{dry})/W_{dry} \times 100$$

Anti-reflux test. The samples were placed on the paper, and a sufficient amount of rhodamine B solution was dropped onto the surface. The sample was then removed at different times to observe whether reflux of the Rhodamine B solution occurred at the bottom of the specimens.

Bacterial growth curves. All the samples were sterilized under UV exposure for 24 h prior to bacterial culture. The original bacterial fluid of *S. aureus* and *E. coli* was inoculated into Luria-Bertani (LB) growth medium for 24 h at 37°C with constant shaking. A typical colony was collected using an inoculation ring into 50 mL of nutrient broth at 37°C for 12 h. The bacterial concentration was 10^7 colony forming units (CFU) per milliliter (CFU mL^{-1}). The resulting *S. aureus* and *E. coli* suspensions were stored in a sterile medical bottle. The suspension was further diluted 10 times to 10^6 CFU mL^{-1}. *S. aureus* or *E. coli* suspensions (4 mL) were added to the centrifuge tube containing the samples. In this assay, the experimental groups were AD-T-1, AD-2 and BC-*g*-PAANa film, and the bacterial suspension was used as the control group. Each sample was repeated three times. Centrifuge tubes were incubated at 37°C for 24-96 h at 180 rpm. During the incubation, the optical density (OD_{600}) value of the above bacterial solutions was measured at different times. Meanwhile, the changes in turbidity of the bacterial culture at different times were observed.

Bacterial morphology. AD-T-1, AD-2 and BC-*g*-PAANa film were placed in the centrifuge tube containing the bacterial suspension (1×10^6 CFU mL^{-1}) and incubated for 24 h. After removal, the samples were gently washed 2-3 times with PBS solution and immersed in 2.5% glutaraldehyde for 30 min. The samples were then dehydrated in aqueous ethanol solutions in a gradient of 30%, 50%, 70%, 80% and 90% (v/v) for 15 min each step, and finally in 100% ethanol for 15 min. Once the samples were sufficiently dried, AD-T-1, AD-2 and BC-*g*-PAANa film were adhered to the sample stage with conductive tape and gilded. The morphology of the bacteria was observed using a field emission scanning electron microscope (FE-SEM, Hitachi S-4800). Bacteria were stained using Photoshop software.

Bacterial penetration assay. For the bacterial penetration assay, sterile pieces of AD-T-1, AD-2 and BC-*g*-PAANa film (with a diameter of 8 mm, and sterilized by UV exposure for 24 h) were placed on an LB agar plate. 10 μL of *S.aureus* and *E. coli* (1 × 10^6 CFU mL^{-1}) suspension were dropped on each surface of the AD-T-1, AD-2, and BC-*g*-PAANa film separately, and incubated at 37°C for 24 h. Finally, the growth of bacteria around the dressing was observed and digital photos were taken.

Cell viability. L929 fibroblasts were resuspended in a complete medium consisting of Dulbecco's modified Eagle medium (DMEM) containing 5% w/v fetal bovine serum (FBS) and 0.5 mL penicillin/streptomycin solution. The cells were then incubated in a CO_2 cell incubator (37°C, 5% CO_2). The complete medium was changed every two days to achieve cell proliferation. The cytotoxicity and proliferation of L929 fibroblasts were measured using a cell counting kit-8 (CCK-8). Specifically, AD-T-1, AD-2 and BC film were cut to the appropriate size and placed at the bottom of 96-well plates. After sterilization by immersion in a mixture of anhydrous ethanol and PBS (75%, v/v) for 12 h, the samples were washed five times with PBS to remove the ethanol. L929 fibroblasts were then inoculated into the sample at a cell density of 2 × 10^4/well, placed in a CO_2 cell culture chamber (37°C, 5% CO_2), and incubated until the cells returned to a normal adherent state. At different time points (day 1, 3, and 5), 10% CCK-8 reagent was added to the appropriate wells, and incubated for 2 h. Cell culture wells inoculated without samples were used as controls. The supernatant obtained was transferred to another 96-well plate. The optical density at 450 nm (OD_{450}) was then measured using an enzyme marker, and cell viability was calculated.

Live/dead staining. A L929 fibroblast suspension (500 μL, 10^5 mL^{-1}) was inoculated into 24-well plates and cultured in a 5% CO_2 atmosphere at 37°C for 24 h. The medium was replaced with AD-T-1, AD-2, and BC film extract (10 mg mL^{-1}) and incubated for 24 h. Subsequently, the cells were stained with a live/dead cell detection kit and visualized using an inverted fluorescence microscope (TI-S, Nikon). Cells seeded in the culture without sample extract served as the control group.

Cytoskeleton staining. The effect of the AD-T-1, AD-2, and BC film on cell viability was further monitored by fluorescence staining. Briefly, L929 fibroblasts were inoculated onto the samples and cultured for 3 days, after which all cell substrates were fixed with 4% paraformaldehyde on ice for 15 min and then washed 3 times with PBS, after which the cells were permeabilized with 0.1% Triton-PBS reagent for 10 min and further washed 3 times with PBS. After completing the above steps, DAPI (Beyotime, China) was used to visualize the nucleus, while Actin-Tracker Green (Beyotime, China) was used to visualize the cytoskeleton. After staining in a dark box for 10 min at room temperature, fluorescence images were captured using a fluorescence microscope (Olympus IX73, Japan) to further identify the distribution of the nucleus and cytoskeleton.

Hemocompatibility. Rabbit blood was centrifuged at 1300 rpm for 10 min to obtain the erythrocytes. The erythrocytes were washed 3 times and diluted to 5% (v/v) with PBS. The BC, AD-2, and AD-T-1were then immersed in PBS and homogenized using a tissue grinder to prepare dispersions of different concentrations (625, 1250, and 2500 μg mL^{-1}). The dispersion liquid (500 μL) and erythrocyte suspension (500 μL) were added to a 2 mL tube, gently mixed, incubated at 37°C for 1 h, and centrifuged at 1300 rpm for 10 min. The supernatant (500 μL) was then transferred separately to new tubes and centrifuged at 13,000 rpm for another 10 min to remove sample particles thoroughly. The as-obtained supernatant (100 μL) was added to a 96-well plate to measure the absorbance at 540 nm using a full wavelength microplate reader (Multiskan GO). 0.1% Triton X-100 was used as the positive control and PBS was used as the negative control. The hemolysis ratio was calculated according to Eq:

$$Hemolysis\ ratio = \frac{A_{\mathrm{p}} - A_{\mathrm{b}}}{A_{\mathrm{t}} - A_{\mathrm{b}}} \times 100\%$$

where A_{p}, A_{t}, and A_{b} are the absorbance values of the supernatants from the sample group, Triton X-100 group and PBS groups, respectively. Each group contains three replicates.

In vivo wound healing. Briefly, Male Sprague Dawley (SD) rats weighing 200-240 g were used. After being anesthetized with pentobarbital (2 wt.%, 1.8 mL kg^{-1}), the dorsal area of the rats was depilated, and 4 full-thickness circular wounds (10 mm in diameter) were created on the upper back of each rat by a disposable 10 mm skin biopsy punch. On each rat, a control wound without treatment was used as the control group. During the observation period, a rat was executed on day 5 and 10. The wound site from the executed rat was harvested in full layer with scissors in conjunction with surrounding tissues, and then soaked in 10% formalin solution. Paraffin embedding was performed as soon as possible for subsequent histological analysis. After surgery, the rats were killed on the spot and taken to a particular area for disposal.

Histological analysis. On the 5th and 10th days after treatment, the wound with surrounding tissue was collected carefully, fixed in 10% paraformaldehyde solution, and embedded in paraffin for routine histological processing. Following a standard protocol, 5 mm thick sections of the tissues were prepared. Hematoxylin-eosin staining (HE) and Masson staining were used to assess morphology, tissue regeneration, and collagen deposition.

Statistical Analysis

All data are represented as the mean ± standard deviation determined by Origin and GraphPad Prism 8 software. Statistical significance of differences between groups was calculated using one-way analysis of variance in Origin software, and all results are labelled $*p < 0.05$.

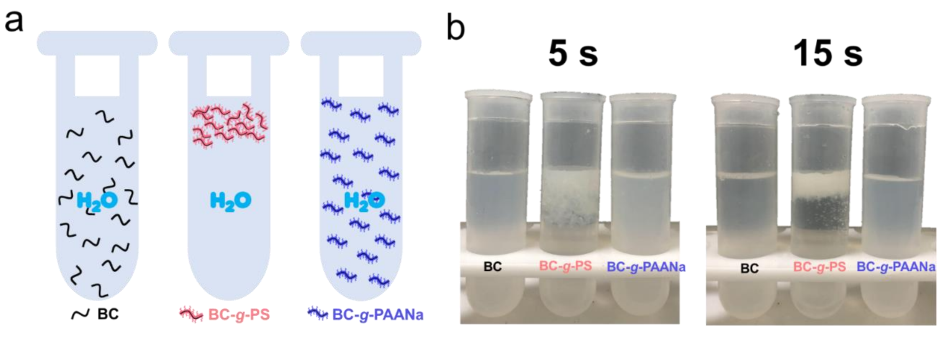

Figure S1 Schematic illustrations (a) and digital photos (b) of the dispersion of BC, BC-*g*-PS, and BC-*g*-PAANa in deionized water.

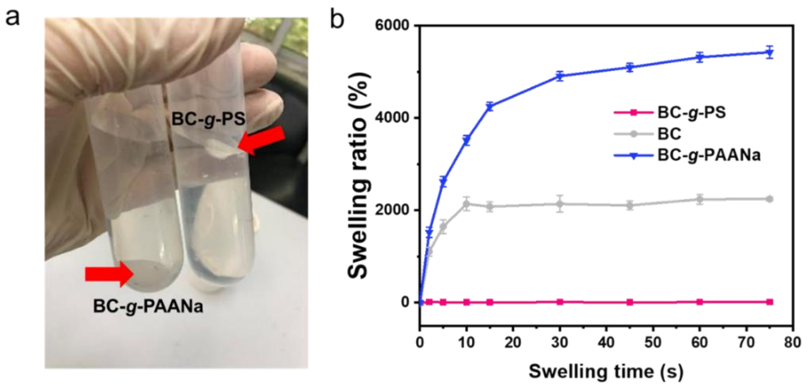

Figure S2 Digital photos of BC-*g*-PS and BC-*g*-PAANa films immersed in deionized water (a). Swelling ratios of BC, BC-*g*-PS, and BC-*g*-PAANa films (b).

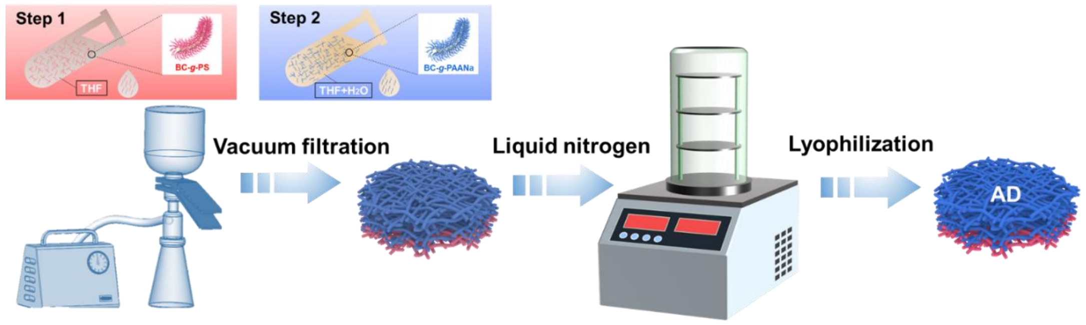

Figure S3 Preparation process of AD, including vacuum filtration and lyophilization.

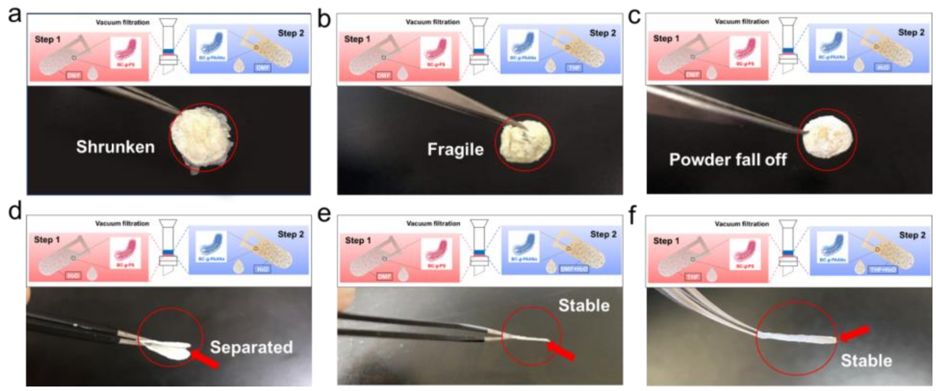

Figure S4 The BC-*g*-PS and BC-*g*-PAANa are dispersed in various solvents and then filtered into the ADs, representing shrunken (a), fragile (b), powder fall off (c), separated (d), and stable states (e, f).

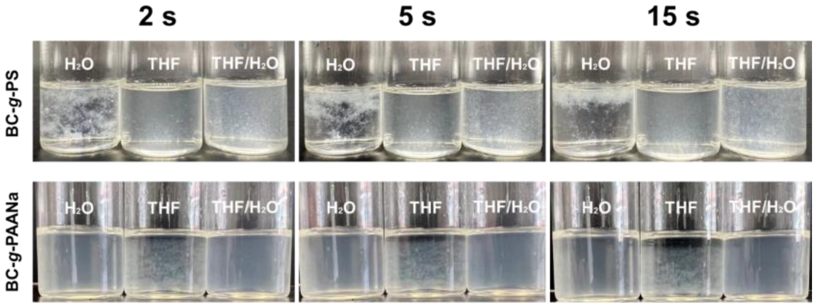

Figure S5 Digital photos of BC-*g*-PS and BC-*g*-PAANa dispersed in deionized water, THF, and a 50% (v/v) aqueous THF solution.

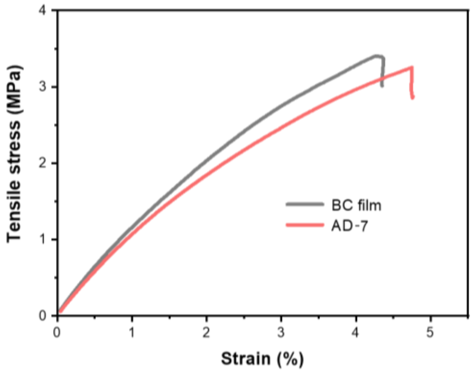

Figure S6 Tensile stress-strain curves of BC film and AD-7.

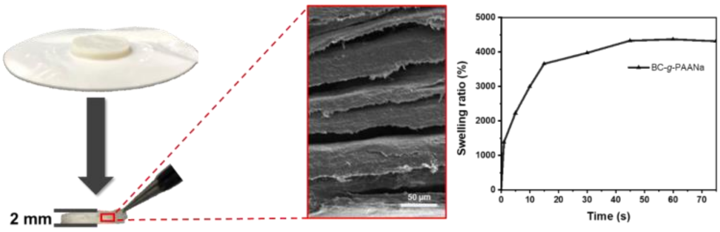

Figure S7 The swelling ratio of BC-*g*-PAANa with a thickness of 2 mm.

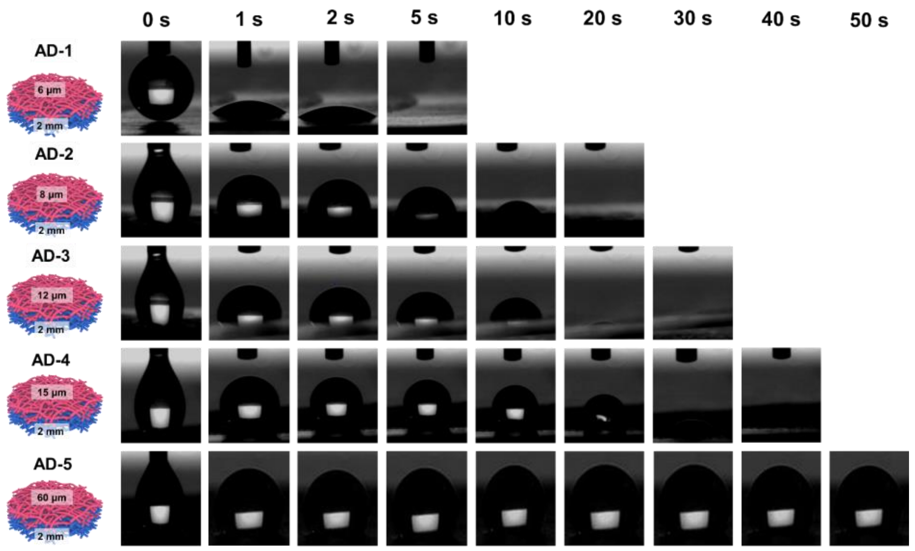

Figure S8 The contact angles of ADs with different thicknesses of BC-*g*-PS at different times.

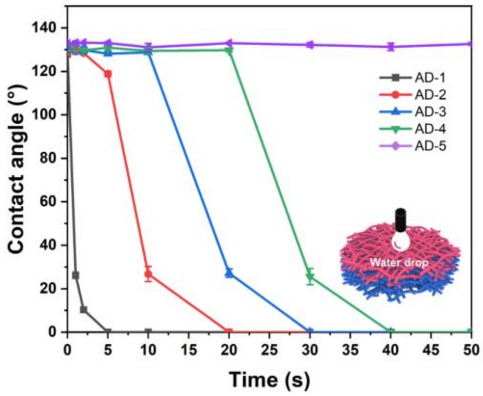

Figure S9 Change in contact angles of ADs with different thickness of BC-*g*-PS over time.

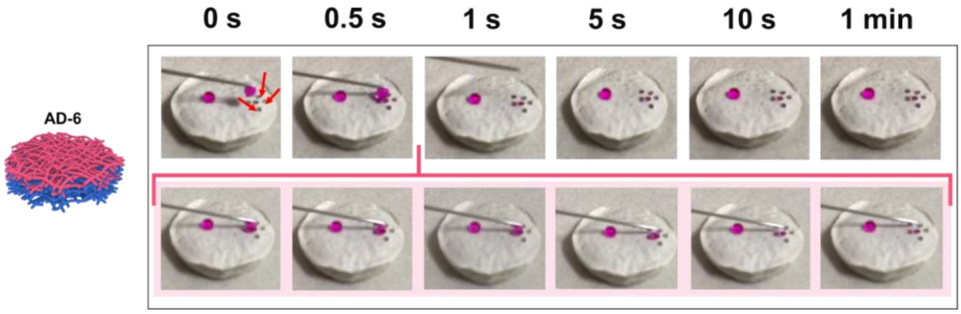

Figure S10 Digital photos of the absorption performance of AD-6.

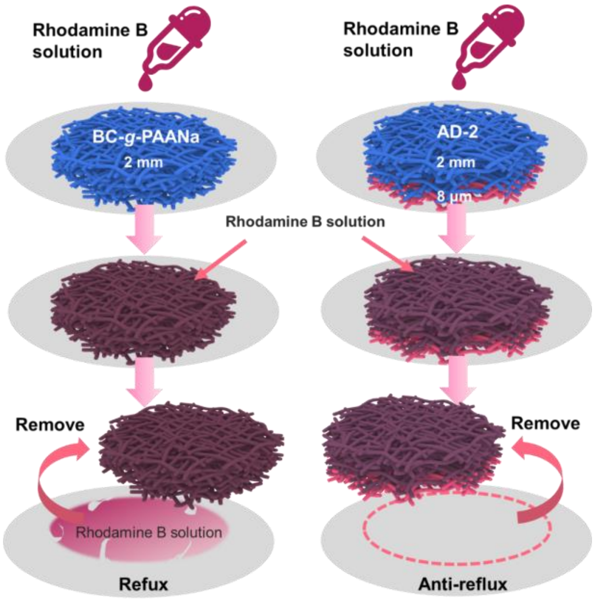

Figure S11 Schematic diagram for anti-reflux performances of BC-*g*-PAANa and AD-2.

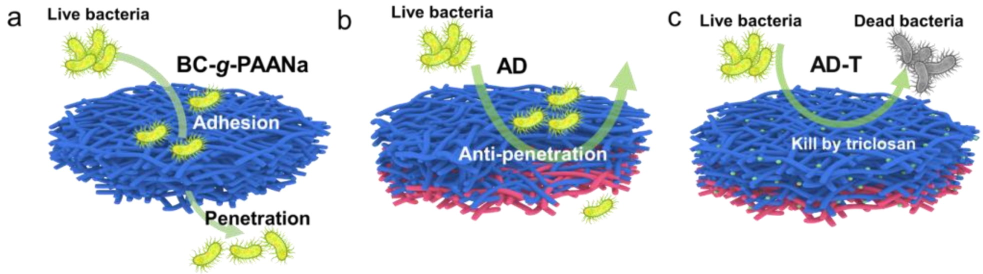

Figure S12 Schematic illustration for bacterial penetration of the BC-*g*-PAANa (a), AD (b), and AD-T (c).

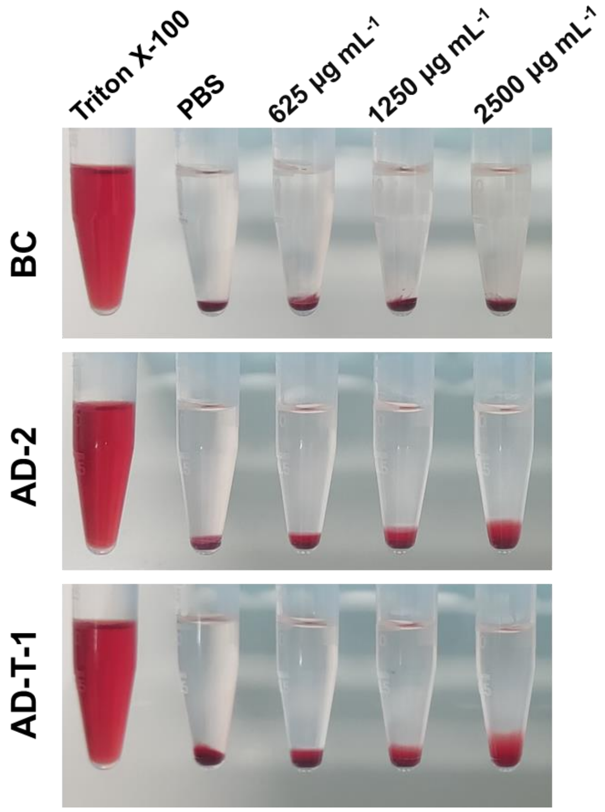

Figure S13 Digital photos of BC, AD-2, and AD-T-1 for hemolysis activity assay, with Triton X-100 serving as positive control, and PBS as negative control.

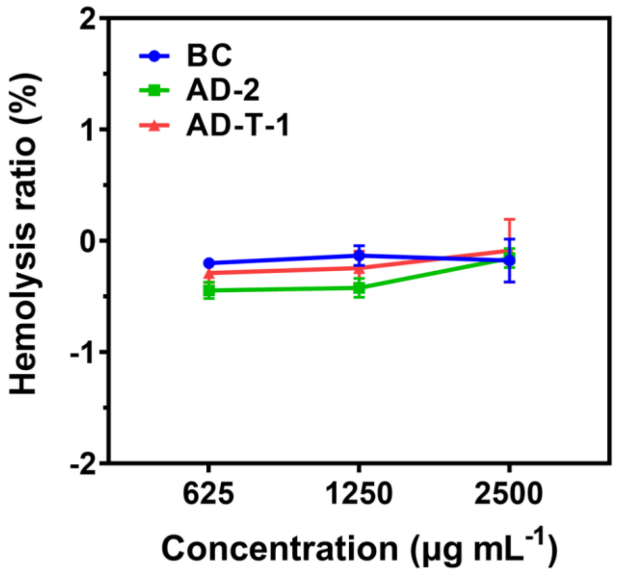

Figure S14 Hemolysis ratios of BC, AD-2, and AD-T-1.

Table S1 Research on wound dressings with hydrophobic component layer.

Structure	Substrate	Hydrophobic layer	Hydrophobic process	Function	Ref.
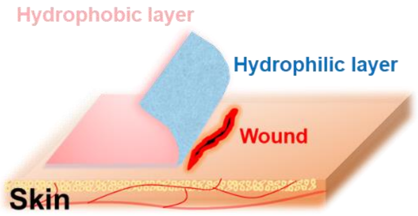	Chitosan sponge	Stearic acid	Coating	Antifouling<br>Anti-bacterial adhesion	Ref. 4
	Cotton fabrics	SiO_2 nanoparticles and ethyl-α-cyanoacrylate superglue	Spraying	Antifouling<br>Anti-blood adhesion	Ref. 5
	Chitosan<br>Poly (vinyl pyrrolidone)<br>Nanocellulose	Stearic acid	Coating	Antifouling<br>Anti-bacterial adhesion	Ref. 6
	Fabrics	Micro- and Nanostructure	Physical modification	Blood repellent	Ref. 7
	CNF-GPTMS-Chitosan Sponge	CNF/VTMS	Blend	Anti-bacterial adhesion<br>Hemostasis	Ref. 8
	PAA/PEI-CMC power	Polycaprolactone	Electrospinning	Anti-bacterial adhesion	Ref. 9
	Cotton Fabrics	Chitosan, gallic acid modified silver nanoparticles<br>and 1H,1H,2H,2H-perfluorodecanethiol	Dip-coating	Antifouling<br>Anti-bacterial	Ref. 10
	Gauze	Paraffin	Dip-coating	Reducing blood loss	Ref. 11
	Composite film	Polylactide, poly(vinyl pyrrolidone), polylactide and poly(ethylene glycol)	Electrospinning	Burns treatment	Ref. 12
	Textile	Poly(dimethylsiloxane), titanium dioxide	Spraying	Blood repellent	Ref. 13

<table>
<tr><td rowspan="2">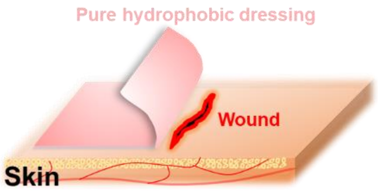
</td><td>Cotton Fabrics</td><td>Silver nitroprusside nanoparticles</td><td>Dip-coating</td><td>Anti-bacterial</td><td>Ref. 14</td></tr>
<tr><td>HA-ADP sponge</td><td>Undecanal-modified chitosan</td><td>Hydrogelation</td><td>Hemostasis</td><td>Ref. 15</td></tr>
<tr><td rowspan="2">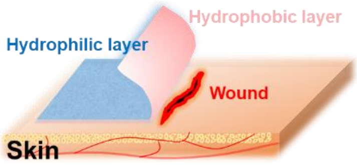
</td><td>Gauze</td><td>Carboxybetaine
ester analogue methacrylate,
poly(ethylene glycol) methacrylate and
aspirin</td><td>Coating</td><td>Antimicrobial
Anti-cell adhesion
Anti-protein adhesion</td><td>Ref. 16</td></tr>
<tr><td>Gauze</td><td>Polydimethylsiloxane,
polytetrafluoroethylene,
carbon nanofibers</td><td>Spraying</td><td>Fast clotting
Minimal adhesion</td><td>Ref. 17</td></tr>
</table>

Table S2 ADs and AD-T-1 consist of BC-*g*-PS and BC-*g*-PAANa in varying proportions.

Sample	BC-*g*-PS		BC-*g*-PAANa	
	V (mL)	Thickness (μm)	V (mL)	Thickness (mm)
AD-1	0.5	6	5	2
AD-2	1	8	5	2
AD-3	2	12	5	2
AD-4	3	15	5	2
AD-5	15	60	5	2
AD-6	30	100	5	5
AD-7	1	8	5	1
AD-8	5	22	1	2
AD-T-1	1	8	5	2

Table S3 Studies on asymmetric dressings with a hydrophilic top layer and a hydrophobic bottom layer.

Hydrophilic layer	Hydrophobic layer	Method	Antibacterial agent	Swelling ratio（%）	Stability between layers	Tensile stress（MPa）	Animal model	Ref.
BC-*g*-PAANa	BC-*g*-PS	Vacuum filtration	Triclosan	~ 4300	Mentioned	~ 3	Acute wound	This work
Medical gauze	PU	Electrospinning	$AgNO_3$	/	/	/	Infected wound	Ref.18
Cellulose acetate	PU	Electrospinning	/	~ 800	/	/	Diabetic wound	Ref.19
ZnCS, $Ca_2ZnSi_2O_7$	PLA	Electrospinning	Zn	~ 100	/	/	Burn wound	Ref.20
QCS/PVA membranes	PCL	Electrospinning	Curcumin	3000-5000	/	/	Diabetic wound	Ref.21
Filter paper	PDMS film	Laser beam and epoxy glue	Amoxicillin	/	Mentioned	/	Infected wound	Ref.22
PLGA/gelatin short fiber	PLGA/PEG/ curcumin	Electrospinning	/	/	/	~ 1	Diabetic wound	Ref.23
Ca-Alg microfibers	PLA	Electrospinning	$AgNO_3$	~ 800	/	/	Infected wound	Ref.24
Gelation fabrics	PVB/PVP	Electrospinning	/	1000-2500	/	~ 6	Diabetic wound	Ref.25
Cotton fabric/TiO_2	PCL	Electrospinning	TiO_2	~ 1000	/	/	Acute wound	Ref.26

References

1. Morandi, G.; Heath, L.; Thielemans, W., *Langmuir* **2009**, *25*, 8280.
2. Dong, R.; Krishnan, S.; Baird, B. A.; Lindau, M.; Ober, C. K., *Biomacromolecules* **2007**, *8*, 3082.
3. Wang, Z.; Crandall, C.; Prautzsch, V. L.; Sahadevan, R.; Menkhaus, T. J.; Fong, H., *ACS Appl. Mater. Interfaces* **2017**, *9*, 4272.
4. D. Liang, Z. Lu, H. Yang, J. Gao, R. Chen, *ACS Appl. Mater. Interfaces* **2016**, *8*, 3958.
5. K. Sasaki, M. Tenjimbayashi, K. Manabe, S. Shiratori, *ACS Appl. Mater. Interfaces* **2016**, *8*, 651.
6. R. Poonguzhali, S. Khaleel Basha, V. Sugantha Kumari, *Int. J. Biol. Macromol.* **2018**, *112*, 1300.
7. V. Jokinen, E. Kankuri, S. Hoshian, S. Franssila, R. H. A. Ras, *Adv. Mater.* **2018**, *30*, e1705104.
8. H. Cheng, D. Xiao, Y. Tang, B. Wang, X. Feng, M. Lu, G. J. Vancso, X. Sui, *Adv. Healthc. Mater.* **2020**, *9*, e1901796.
9. C. Hui, Y. Gao, B. Yan, L. Ding, T. Sun, Z. Liu, S. Ramakrishna, Y. Long, J. Zhang, *Chem. Eng. J.* **2023**, *464*, 142458.
10. G. Liu, J. Xiang, Q. Xia, K. Li, T. Lan, L. Yu, *Cellulose* **2018**, *26*, 1383.
11. T. Zhu, J. Wu, N. Zhao, C. Cai, Z. Qian, F. Si, H. Luo, J. Guo, X. Lai, L. Shao, J. Xu, *Adv. Healthc. Mater.* **2018**, *7*, e1701086.
12. W. Li, Q. Yu, H. Yao, Y. Zhu, P. D. Topham, K. Yue, L. Ren, L. Wang, *Acta Biomater.* **2019**, *92*, 60.
13. J. Liu, L. Ye, Y. Sun, M. Hu, F. Chen, S. Wegner, V. Mailander, W. Steffen, M. Kappl, H. J. Butt, *Adv. Mater.* **2020**, *32*, e1908008.
14. B. R. Rao, R. Kumar, S. Haque, J. M. Kumar, T. N. Rao, R. Kothapalli, C. R. Patra, *ACS Appl. Mater. Interfaces* **2021**, *13*, 10689.
15. Y. Liu, H. Niu, C. Wang, X. Yang, W. Li, Y. Zhang, X. Ma, Y. Xu, P. Zheng, J. Wang, K. Dai, *Bioact. Mater.* **2022**, *17*, 162.
16. F. Ji, W. Lin, Z. Wang, L. Wang, J. Zhang, G. Ma, S. Chen, *ACS Appl. Mater.

Interfaces **2013**, *5*, 10489.

17. Z. Li, A. Milionis, Y. Zheng, M. Yee, L. Codispoti, F. Tan, D. Poulikakos, C. H. Yap, *Nat. Commun.* **2019**, *10*, 5562.
18. Shi, L.; Liu, X.; Wang, W.; Jiang, L.; Wang, S., *Adv. Mater.* **2019**, *31*, e1804187.
19. Bao, F.; Pei, G.; Wu, Z.; Zhuang, H.; Zhang, Z.; Huan, Z.; Wu, C.; Chang, J., *Adv. Funct. Mater*. **2020,** *30*, 2005422.
20. Zhang, Z.; Li, W.; Liu, Y.; Yang, Z.; Ma, L.; Zhuang, H.; Wang, E.; Wu, C.; Huan, Z.; Guo, F.; Chang, J., *Bioact. Mater.* **2021**, *6*, 1910.
21. Zhang, K.; Jiao, X.; Zhou, L.; Wang, J.; Wang, C.; Qin, Y.; Wen, Y., *Biomaterials* **2021**, *276*, 121040.
22. Xu, B.; Li, A.; Wang, R.; Zhang, J.; Ding, Y.; Pan, D.; Shen, Z., *Adv. Funct. Mater.* **2021**, *31*, 2105265.
23. Qian, S.; Wang, J.; Liu, Z.; Mao, J.; Zhao, B.; Mao, X.; Zhang, L.; Cheng, L.; Zhang, Y.; Sun, X.; Cui, W., *Small* **2022**, *18*, e2200799.
24. Zhang, H.; Sun, L.; Guo, J.; Zhao, Y., *Research* **2023**, *6*, 0129.
25. Zhou, L.; Xu, P.; Dong, P.; Ou, X.; Du, X.; Chen, Y.; Zhang, X.; Guo, W.; Gao, G., *Chem. Eng. J.* **2023**, *457*, 141108.
26. Pi, H.; Xi, Y.; Wu, J.; Hu, M.; Tian, B.; Yang, Y.; Wang, R.; Zhang, X., *Chem. Eng. J.* **2023**, *455*, 140853.
